# Ultra Rapid Object Categorization: Effects of Level, Animacy and Context

**DOI:** 10.1371/journal.pone.0068051

**Published:** 2013-06-28

**Authors:** Maren Praß, Cathleen Grimsen, Martina König, Manfred Fahle

**Affiliations:** 1 Center for Cognitive Science, Human Neurobiology, Bremen University, Bremen, Germany; 2 The Henry Wellcome Laboratories for Vision Sciences, City University London, London, United Kingdom; National Institute of Mental Health, United States of America

## Abstract

It is widely agreed that in object categorization bottom-up and top-down influences interact. How top-down processes affect categorization has been primarily investigated in isolation, with only one higher level process at a time being manipulated. Here, we investigate the combination of different top-down influences (by varying the level of category, the animacy and the background of the object) and their effect on rapid object categorization. Subjects participated in a two-alternative forced choice rapid categorization task, while we measured accuracy and reaction times. Subjects had to categorize objects on the superordinate, basic or subordinate level. Objects belonged to the category animal or vehicle and each object was presented on a gray, congruent (upright) or incongruent (inverted) background. The results show that each top-down manipulation impacts object categorization and that they interact strongly. The best categorization was achieved on the superordinate level, providing no advantage for basic level in rapid categorization. Categorization between vehicles was faster than between animals on the basic level and vice versa on the subordinate level. Objects in homogenous gray background (context) yielded better overall performance than objects embedded in complex scenes, an effect most prominent on the subordinate level. An inverted background had no negative effect on object categorization compared to upright scenes. These results show how different top-down manipulations, such as category level, category type and background information, are related. We discuss the implications of top-down interactions on the interpretation of categorization results.

## Introduction

In object categorization objects are classified corresponding to common characteristics, a process which depends both on perceptual and higher cognitive processing stages. The perceptual part of object categorization includes early sensory processing (contrast detection, contour integration, color processing and segmentation from background), and complex visual processing, like object detection and coarse classification to certain categories (e.g. faces, objects, scenes). Cognitive aspects of object processing are associated with semantic interpretation, classification, memorization and lexical processing of an object. Top-down influences, such as expectation, attention, context and expertise, are believed to facilitate object recognition.

The aim of investigating perceptual object categorization is to understand how objects and scenes are represented in early and higher visual cortices. It is known that the visual system is extremely fast and accurate in recognizing complex natural scenes and objects [1], [2]. It is assumed that a first sweep of feedforward information is sufficient to discriminate whether or not an object is present in a scene [3], [4] and that top-down processes can speed up response times [2], [5], [6]. Key visual features of an object (such as eyes, mouth and limbs of an animal) are crucial, while color information plays a minor role in rapid categorization [7]. Studies have claimed that the rapid detection of an object in a scene might be pre-attentive [8]–[10], but this view has been challenged recently, showing that scene perception requires attention [11]. If demands on object categorization are more complex, such as identification of an object, higher stages of cognitive processing are required [12]–[15]. These processes are beyond pure object detection and include a more detailed analysis of the object and its semantic interpretation. Aspects influencing object categorization in a top-down manner are, among others, spatial and feature-based attention, the likelihood of an object being present, expertise, the level of abstraction and, thus, the amount of information necessary to analyze the object, the category of an object, e.g. living or non-living, and the contextual information an object co-occurs with. This study combines perceptual categorization processes (rapid visual object categorization) and the influence of top-down processes (here: level of abstraction, animacy and contextual background) that potentially impact on object categorization.

### Levels of Abstraction

The same object can be categorized at different levels of abstraction, for example at a general, superordinate (e.g. animal), a basic (e.g. cat) or a subordinate (e.g. Siamese cat) level [16]. As the object itself is always the same, the effect of level is clearly not a perceptual one, but rather a cognitive process where information is evaluated corresponding to task demands. It is a matter of discussion what the entry level of categorization is. Some studies state an advantage for the basic level (e.g. “cat”) over both the more general category “animal” (superordinate level) and the more precise category “Siamese cat” (subordinate level) [16]–[18]. It is assumed that the basic level is processed before the superordinate and subordinate level. Other studies found evidence against the basic level advantage [19]–[21]. It has been suggested that the advantage for basic level scenes diminishes if stimulus processing time is limited [18], [19], [21]. In a go/no-go paradigm subjects responded faster and more accurate to the category “animal” than to the basic level category “bird” or “dog” [19]. According to a coarse-to-fine account, coarse information is important for global image features and fine information for local image features. Thus, the more specific an object is categorized, the finer grained the perceptual information about that object should be [22], [23]. This hypothesis argues against an advantage for the basic level because for basic level categorization finer perceptual information is needed than for superordinate categorization. It remains elusive under what circumstances the basic-level advantage occurs. The question arises whether the loss of the basic level advantage is a consequence of rapid categorization processes and whether it can be replicated in other test situations using rapid categorization tasks. We tested the occurrence of the basic-level advantage in a two alternative forced choice (2-AFC) task. Thus, for each stimulus presentation a response was given which allowed for the categories to be compared directly. Categories were more similar than in the study of Macé et al. to assess the effect of level under more controlled stimulus conditions. Based on the results of Macé and colleagues we hypothesized a behavioral advantage for the superordinate over the basic level, probably due to limited processing time in rapid categorization.

### Animate vs. Inanimate categories

Objects can also be classified into living and non-living categories. Different processing mechanisms for animate and inanimate object categories have been suggested by behavioral, functional and neuropsychological studies [24]–[26]. Animate/inanimate categories were found to engage different neural subsystems in the brain [13], [27], [28]. This may lead to faster and more accurate responses for either an animate or an inanimate category [29]–[32]. These animacy effects might be derived from perceptual differences between object categories. It has been shown, for example, that animate categories depend on different spatial frequencies than categorization of non-animals [33], [34]. Additionally, cognitive processes, like different functionality and specialization of the object, may account for animacy effects, (e.g. tools automatically recruit action related circuits; [14]).

How animate and inanimate objects affect behavior and whether a behavioral advantage for either category exists is still an open question. Here we use animate and non-manipulable inanimate categories at different levels of abstraction in order to investigate whether animate categories yield better performance than non-manipulable inanimate categories. This suggestion is based on the *sensory/functional account theory*, which assumes that objects are represented according to their information content and functionality [35], [36]. Following this theory, manipulable artifacts, such as tools, have a behavioral benefit over animate categories, because faster, action related neural circuits are engaged during processing. On the other hand, processing of non-manipulable artifacts, such as vehicles, depend more on perceptual than on functional properties. This would lower the behavioral benefit and lead to similar or even worse performance, compared to animate categories.

It is unknown whether animate or inanimate objects are categorized differently at different levels of abstraction. We therefore explored the effect of animate and inanimate categories at the superordinate, basic and subordinate level. It was hypothesized that a similar animacy effect should occur at each level of abstraction.

### Background

The context in which an object occurs may be crucial for its recognition. Objects usually co-occur with a contextual frame which is perceptually and semantically congruent with the object. A bird belongs to a tree, a car on a street and a computer on a desk. Semantically congruent contextual associations have been suggested to facilitate both bottom-up and top-down object recognition [5], [37]–[39]. Furthermore, in rapid categorization subjects are better and faster detecting objects in a semantically congruent context [32], [40]. Another factor may be the physical relationship between an object and its background. That is, object-background proportions such as size, position and orientation may influence object categorization [41]–[43]. It remains an open question whether or not such perceptually (physically) incongruent background information affect rapid object categorization processes and whether such physical manipulations differ from semantically manipulations. In this study the background information was varied in terms of orientation (upright vs. inverted context), while keeping the level and category constant. This allows for the investigation of background manipulations while controlling for object types and task demands. A “no background” (gray background) condition was used to compare categorization performance between isolated objects and objects embedded in a complex natural scene background. An inverted scene is believed to hamper processing of the object categorization, thus leading to higher error rates and slower reaction times.

Several studies have shown that the level, animacy and background of objects influence categorization processes, but the effects and interactions of these higher cognitive processes on perceptual categorization remain unclear. The experiments of the current study address the question of how perceptual and cognitive task manipulations influence rapid categorization. The study was driven by two main motivations: investigating (1) aspects of perceptual stages of object processing (bottom-up mechanisms) using rapid object categorization and speeded response times and (2) top-down mechanisms using manipulations of category level, animacy and background. The brief presentation time of stimuli (30 msec) was chosen to keep high-level factors known to affect object categorization, such as expectations, intentions and expertise [15], [44]–[46], to a minimum. An image, belonging to one of two given categories, was presented briefly and responses were given as quickly as possible via a button press in a two-alternative forced choice. Objects could either belong to a living (animal) or a non-living (vehicle) category and each object was presented on three different backgrounds (gray, upright and inverted). Upright and inverted backgrounds consisted of complex natural scenes and were semantically congruent with the given object. The task was to categorize objects at the superordinate, basic and subordinate level of abstraction in separate runs (e.g. animal, dog and St. Bernard, respectively). Importantly, no verbal response was necessary, in order to exclude lexical processes.

## Materials and Methods

### Ethics Statement

The study was approved by the local ethics committee of the University of Bremen (IACUC permit numbers and IRB name: n/a) and is in accordance with the Declaration of Helsinki. All subjects gave informed written consent.

### Participants

Sixteen healthy participants (8 male, 8 female) volunteered for the study. The age ranged between 18–31 years (mean: 25±4 years). Subjects had normal or corrected-to-normal visual acuity and no ophthalmological or neurological disorders (self-report). They were informed about the study and received course credits for participation. Subjects were non-experts but familiar with the given categories.

### Experimental Design and Procedure

**Figure 1:**
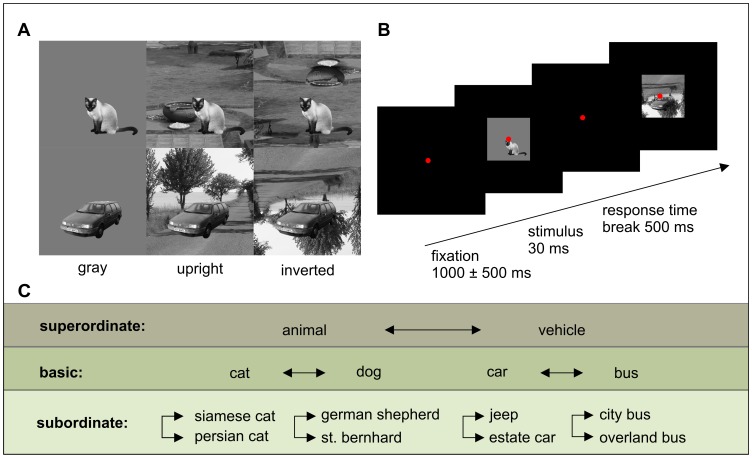
Illustration of stimuli and experimental design. (A) Two example objects on different context conditions. (B) Trial sequence. (C) Schematic of tested categories on each level of categorization. The arrow indicates pairs of categories that were tested against each other.

Three different category-levels where examined, namely the superordinate, basic and subordinate levels (Figure 1). The superordinate level contained the categories animal vs. vehicle. The basic level included the categories dog vs. cat and car vs. bus, while the subordinate level included the categories German Shepherd vs. St. Bernard, Siamese cat vs. Persian cat, estate car vs. Jeep and overland bus vs. city bus. Each pair of categories was presented in a single run, resulting in seven independent runs. In each run 180 trials were presented, including two categories with 30 objects; each object was presented on three different backgrounds (gray, upright natural scene and inverted natural scene, Figure 1a). The sequence of stimulus presentations was counterbalanced throughout each run.

A trial started with a fixation time of 1000 msec (±500 msec jitter), followed by stimulus presentation for 30 msec and an unlimited response time (Figure 1b). The stimuli were presented centrally with a size of 9.4° of visual angle. The trial ended with a 500 msec break after the subject´s response and before a new trial started. The screen was dark and a red fixation point (12 arcmin) was on the screen at all times. Participants had to indicate to which of the two categories the presented stimulus belonged through button press, irrespective of the background the object was presented on (two-alternative forced choice task). Response buttons were held in the left and right hand. Participants were instructed to respond as quickly and as accurately as possible and an auditory feedback was applied after false responses.

### Stimuli and Apparatus

Stimuli consisted of black and white photographs of natural scenes, which were taken from an image hosting website (flickr.com) and which had no copyright restrictions. The target objects were cropped out and pasted on a gray, an upright and an inverted natural scene background (on the same position). Edges of objects were smoothed to reduce pasting effects. All target objects had roughly the same size (120 pixels) and covered one third of the total image (350×350 pixels). For the subordinate level, each category consisted of 30 different objects and each object was pasted on three different backgrounds (gray, upright natural scene and inverted natural scene), resulting in 90 images for each category. In total, 720 images were created for the eight categories of the subordinate level. Both, superordinate and basic level image sets were composed of the subordinate image set. For the basic level category “dog”, half of the images derived from the German Shepherd set and the other half from the St. Bernard set. The other three basic level categories derived from their corresponding subordinate categories likewise. The image sets of the superordinate level consisted of equal portions of the appropriate subordinate image sets. The categories German Shepherd, St. Bernard, Siamese cat and Persian cat composed the superordinate category “animal” and the categories estate car, Jeep, overland bus and city bus composed the category “vehicle”. With respect to a balanced composition of images for basic and superordinate level sets, different images were taken from the original subordinate set. This was applied to avoid memory effects due to repetitive presentations of identical images. Participants were divided into two groups, which were presented with different image sets of the basic and superordinate level. Each participant was presented with all subordinate level images.

Stimuli were presented in a dark room on a 20” CRT monitor (SAMSUNG Syncmaster 1100 MB; refresh rate 100 Hz and 1280×1024-pixel resolution) at a viewing distance of 60 cm, which was sustained by a chinrest. The stimulus presentation was conducted with an in-house software on a standard PC.

### Analysis

Reaction times (RT) and the proportion of correct responses (%-correct) were calculated. Statistical analysis was performed by using PASW statistics 18 (version 18.0.0). To analyze the obtained results repeated measures Analysis of Variance (ANOVA), post hoc pairwise comparisons and paired t-tests were conducted. In case sphericity was violated in the repeated measures ANOVA, a Greenhouse-Geisser correction was applied. Error bars of graphs represent normalized confidence intervals according to an approach by Cousineau (2005) and Morey (2008) [47], [48].

## Results

This study investigated the influence of different a) levels of abstraction, b) animacy and c) background on ultra rapid object categorization. The percentages of correct responses as well as the reaction times were analyzed with a 3 (level: superordinate, basic, subordinate)×2 (animacy: animal, vehicle)×3 (background: gray, upright, inverted) repeated-measures Analysis of Variance (ANOVA). Results are shown in Table 1.

**Table 1:** Mean reaction times (ms) and %-correct for categorization of different levels, animacy and context.

Level	Animacy	Context	Reaction time		%-correct	
			Mean	SE	Mean	SE
superordinate	animal	gray	459	49	94.2	6.3
		upright	455	58	91.5	5.8
		inverted	487	41	94.8	3.2
	vehicle	gray	469	61	92.5	7.3
		upright	488	57	95.8	4.5
		inverted	473	59	94.2	4.3
basic	animal	gray	519	40	90.8	7.3
		upright	465	39	89.6	3.9
		inverted	546	47	87.8	6.1
	vehicle	gray	479	35	94.3	4.6
		upright	544	45	93.5	4.5
		inverted	481	40	94.3	4.2
subordinate	animal	gray	539	52	88.8	6.2
		upright	559	41	86.6	5.9
		inverted	564	43	85.8	5.2
	vehicle	gray	571	47	85.8	3.8
		upright	561	53	80.9	5.2
		inverted	568	42	80.2	4.7

### Main Effects of Top-down Manipulations

#### Accuracy

**Figure 2:**
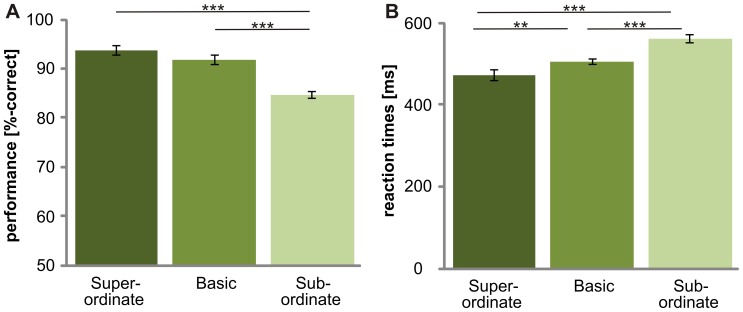
Influence of “level” on ultra-rapid object categorization. For each level (superordinate, basic and subordinate) the mean performance (A) and reaction times (B) are shown. Error bars represent the normalized 95% confidence intervals of the mean (Cousineau-Morey approach [47], [48]). *p<.05 **p≤.01 ***p≤.001.

**Figure 3:**
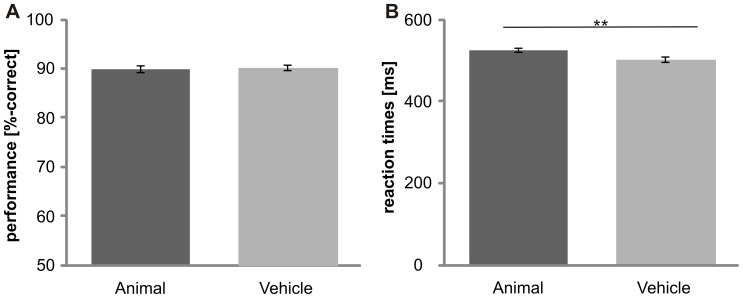
Influence of “animacy” on ultra-rapid object categorization. Performance (A) and reaction times (B) for animal and vehicle category are shown. Error bars represent the normalized 95% confidence intervals of the mean (Cousineau-Morey approach [47], [48]). *p<.05, **p≤.01, ***p≤.001.

**Figure 4:**
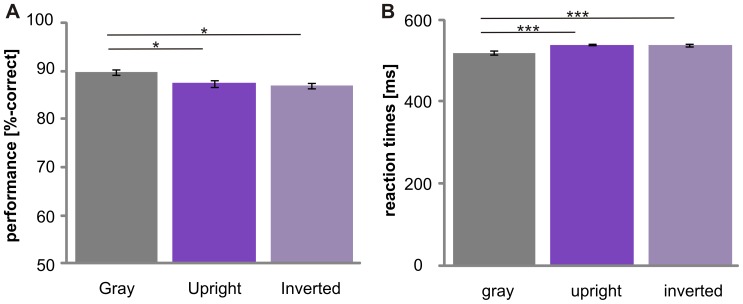
Effect of context on object categorization. The responses (percentage correct (A) and reaction times (B)) are shown for different context conditions. Objects were embedded in either a gray, upright or inverted context. Error bars represent the normalized 95% confidence intervals of the mean (Cousineau-Morey approach [47], [48]). *p<.05, **p≤.01 ***, p≤.001.

Subjects showed different accuracies at the superordinate level, the basic level and the subordinate level (F_(2,30)_ = 98.8, p<0.001; Figure 2a). Performance was best at the superordinate level and was decreased at the basic (p≤0.05) and subordinate levels (p<0.001). Accuracy was higher at the basic level than at the subordinate level (p<0.001). No significant difference was found for animal and vehicle categorization (F_(1, 15)_ = .1, p>.05; Figure 3a). Subjects were much better at categorizing objects on a gray background than objects on complex natural scenes (p≤.05; Figure 4a). No significant difference was obtained between the “upright background” and “inverted background” conditions (p>.05). Thus, a gray background facilitated object categorization but manipulating the orientation of a complex background had no influence on the categorization performance (F_(2, 30)_ = 6.6; p = 0.004).

#### Reaction Times

Reaction times were different between superordinate, basic and subordinate level (F_(1,21)_ = 68.8, p<0.001; due to violation of sphericity Greenhouse-Geisser corrected; Figure 2b). Object categorization at the superordinate level was significantly faster than at the basic (p≤0.05) and subordinate (p<0.001) levels. Reaction times were faster at the basic level than at the subordinate level (p<0.001). Thus, no basic level advantage was observed. Animate categories were processed slower than inanimate categories (Figure 3b). Reaction times for animals (523±41ms) were significantly slower than the reaction times for vehicles (502±41ms; F_(1,15)_ = 14.2, p<.01). These results suggest that processing speed, as measured by reaction time, is influenced by animacy, but performance is not. The background had an effect on categorization (F_(2,30)_ = 39.3; p<0.001): Objects on a gray background were categorized faster than objects embedded in scenes (p≤.05). No difference was found between upright and inverted scenes (p>.05). This demonstrates that a lack of a complex background facilitates object recognition (Figure 4b).

### Interaction of Level and Animacy

#### Accuracy

**Figure 5:**
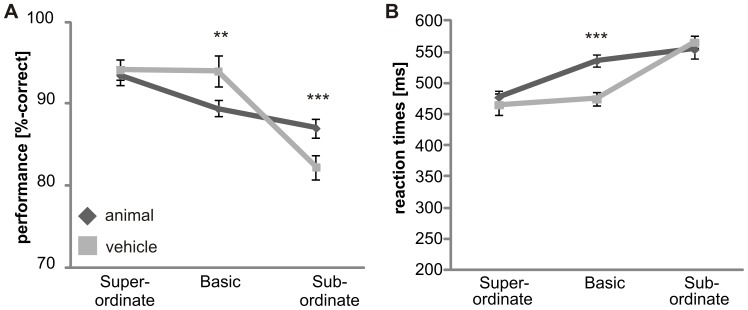
Interaction of level of categorization and different categories (animal and vehicle). Performance (A) and reaction times (B) for animal and vehicle category on each level of categorization are shown. Error bars represent the normalized 95% confidence intervals of the mean (Cousineau-Morey approach [47], [48]). *p<.05, **p≤.01, ***p≤.001.

There was a significant interaction between level and animacy (F_(2, 30)_ = 20.6; p<0.001). Animals and vehicles were thus compared at each category level, showing that at the basic level subjects showed higher accuracy for vehicles than for animals (t_(15)_ = −3.9, p≤0.001). At the subordinate level higher accuracy was observed for the animal category (t_(15)_ = 4.4, p<0.001) and at the superordinate level, no significant difference was obtained for animal and vehicle categories (t_(15)_ = −.9, p≥.05). These results indicate that the categories used in this study reveal different performance patterns at different category levels (significant results shown in Figure 5, vertical comparison).

We compared the category levels within each category (horizontal comparisons in Figure 5). Subjects showed highest accuracy for animals at the superordinate level but performance was significantly lower at the basic (t_(15)_ = 3.1, p<.01) and subordinate (t_(15)_ = 6.1, p<.001) level, both to a similar degree. For the vehicle category, we observed a different response pattern. Here, subjects obtained the same accuracy at the superordinate and basic level (t_(15)_ = .3, >.05) and accuracy was decreased at the subordinate level compared to the superordinate (t_(15)_ = 15.6, p<001) and basic levels (t_(15)_ = 15.8, p<001).

#### Reaction times

Subjects responded with the same speed to animals and vehicles at the superordinate (t_(15)_ = 1.9, p = ≥.05) and subordinate (t_(15)_ = −1.3, p≥.05) level. However, at the basic level they were significantly faster for vehicles than animals (t_(15)_ = 8.6, p<0.001; F_(2, 30)_ = 20.6; p<0.001). This interaction reveals that the benefit for vehicles is completely driven by the basic level.

Within the category “animal”, subjects showed the fastest reaction times at the superordinate level, as compared to the basic (t_(15)_ = −7.6, p<.001) and subordinate (t_(15)_ = −7.2, p<.001) level. Subjects responded significantly slower for animals on the subordinate level than on the basic level (t_(15)_ = −2.7, p = .015). Within the category “vehicle” subjects showed no difference in reaction times on the superordinate and basic level (t_(15)_ = −.9, p>.05), but reaction times were significantly decreased at the subordinate level compared to the superordinate (t_(15)_ = −8.7, p<001) and basic level (t_(15)_ = −18.3, p<001).

These results suggest that the living and non-living categories tested in this study, yield different performance patterns across the levels. Especially at the basic level, subjects showed a behavioral advantage for vehicles over animals.

### Interaction of Level and Background

#### Accuracy

**Figure 6:**
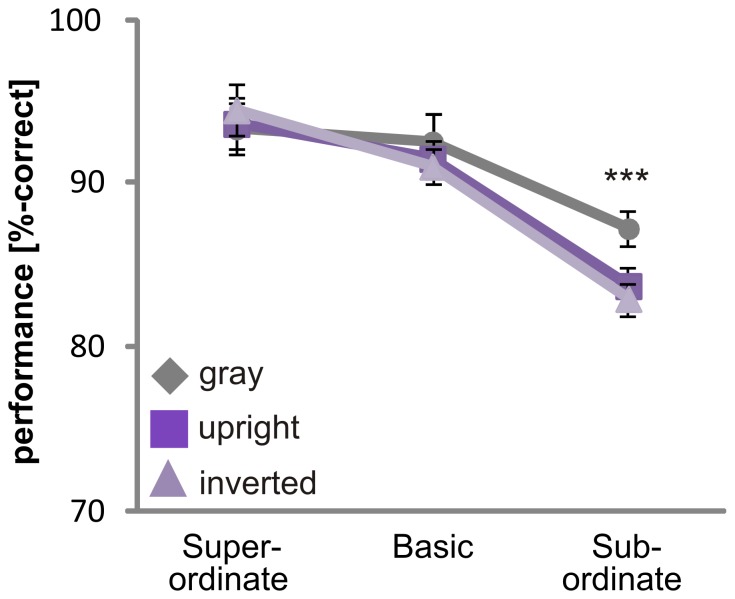
Interaction of level and context. Higher accuracy was obtained for the gray context condition at the subordinate level. Error bars represent the normalized 95% confidence intervals of the mean (Cousineau-Morey approach [47], [48]). *p<.05, **p≤.01, ***p≤.001.

Subjects were better at categorizing objects on a gray background than objects on a natural upright (t_(15)_ = 5.1, p<.001) or inverted (t_(15)_ = 7.3, p<.001) background only at the subordinate level, as revealed by a significant interaction between level and context for percentage correct (F_(2, 33)_ = 5.2, p = .01; Greenhouse-Geisser corrected; Figure 6). This is at odds with the reaction time data, where responses were faster for objects on gray backgrounds at each level (see section “Main effects of top-down manipulations”).

### Interaction of Animacy and Background

#### Reaction times

**Figure 7:**
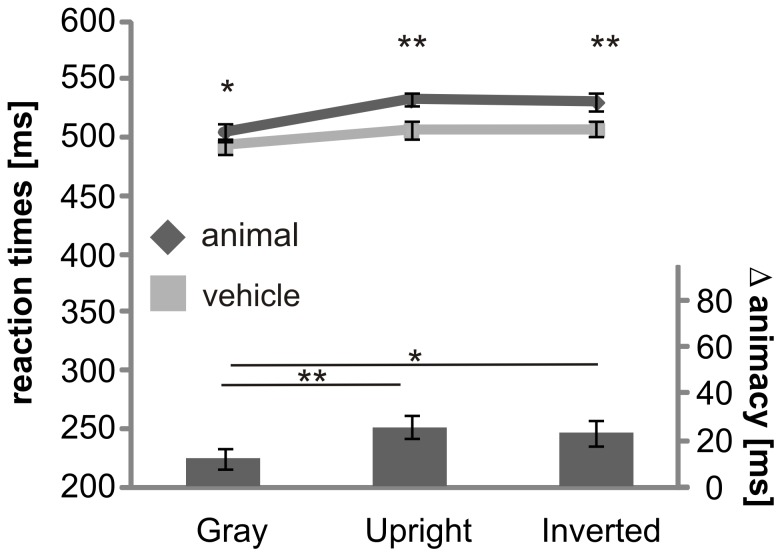
Interaction of animacy and context. Reaction times for animal and vehicle categories in all three context conditions are shown. The bar graphs represent the calculated difference between animal and vehicle condition. Error bars represent the normalized 95% confidence intervals of the mean (Cousineau-Morey approach [47], [48]). *p<.05, **p≤.01, ***p≤.001.

Reaction times were significantly slower for animate than vehicle categories in all background conditions (gray: t_(15)_ = 2.2, p = .047; upright: t_(15)_ = 4.3, p = .001; inverted: t_(15)_ = 3.9, p = .001), but subjects showed even slower response times when animals were on complex backgrounds (F_(2, 30)_ = 6.4, p = .005). This was evaluated by calculating the difference of reaction times between animate and vehicle categories for each background condition. The difference between animate and vehicle conditions on gray backgrounds was smaller than for upright backgrounds (t_(15)_ = −3.5, p = .003) and inverted backgrounds (t_(15)_ = −2.9, p = .01). A higher difference indicated slower reaction times for the animate category (Figure 7).

## Discussion

In this study we investigated aspects of three different top-down parameters on a perceptual object categorization task. We manipulated the level (superordinate, basic and subordinate), animacy (animal, vehicle) and background (gray, upright, inverted) of objects and found that all of these manipulations affected rapid categorization processes. Importantly, the top-down processes interacted with each other specifically, such that their effect on the categorization task depended on the combination of parameters. This has an impact on the interpretation of categorization results.

### Levels of Categorization

Regarding the influence of level on object categorization, we found a clear advantage for the superordinate level over the basic and subordinate levels and hence, no basic level advantage. Because many studies have found an advantage for the basic level, this level was often thought to be the entry level of categorization [16], [49]. Subjects appeared to have a behavioral benefit for the category “dog” over the category “animal” or the category “St. Bernard”. In contrast, we did not find a basic level advantage in a two-alternative-forced choice rapid object categorization task, where categories were controlled for similarity. Recent evidence suggests that the basic level advantage does not occur for limited response times [18] and for rapid object and scene categorization [19]–[21], [50], [51]. Compared to previous rapid categorization studies, categories in the current study were less heterogeneous because only a subset of existing categories that were as similar as possible perceptually were used. This was done deliberately to minimize any potential confounds caused by large differences in the visual features of the objects, both within and between categories. Nevertheless, visual features at the superordinate level (e.g. dog vs. car) differed to a greater extent than visual features at the basic level (e.g. dog vs. cat). This may explain the advantage of the superordinate level simply because it was more heterogeneous than the basic level. Macé and colleagues (2009) obtained similar effects as presented here: they found an advantage for the detection of an animal (superordinate level) over the detection of a bird or a dog in a natural scene (basic level). Thus, the effect of better superordinate categorization performance seems to occur irrespective of chosen categories. Furthermore, patient studies reveal a selective impairment of categorization on the basic level, while leaving the superordinate level unaffected [18]. This challenges the notion of a perceptual mechanism, where, at a first step, the basic level is processed as a general entry level. Rather, the basic level advantage might occur in certain test situations, e.g. for tasks with main emphasis on cognitive categorization with long presentation times (e.g. word-picture combination, [18]) or if verbal responses are required. Objects are usually named on their basic level more frequently than at their superordinate or subordinate level, which yields to faster retrieval of basic level categories [16], [17], [52]. In the current study processing time was limited due to very short image presentation times, and responses were given via a button press. It might be possible that, depending on task requirements (naming or button press), the category levels are processed differently or that the processing mechanisms are tapped at different processing stages, thus, leading to different outcomes. Our results reveal no advantage for the basic level and this strengthens the assumption of a coarse to fine grained analysis of perceptual object information [19], rather than a beneficial processing for basic level object features. The advantage for the basic level seems to depend on higher cognitive processes, which may be strongly influenced by task demands [53].

### Animacy in Object Recognition

One topic which is still under debate is the question whether a behavioral benefit exists for inanimate over animate object categories. According to the *sensory/functional account*, an advantage for animate objects over non-manipulable inanimate objects was expected in the current study. However, the reversed effect was observed. Both categories revealed similar error rates, but reaction times were significantly faster for the inanimate category (vehicles) than for the animate category (animals). If the levels of categorization are taken into account, it becomes clear that the advantage of inanimate categories is only observed at the basic level, with the vehicles category displaying higher percentage correct and faster reaction times. Interestingly, at the basic level there was a significantly higher accuracy for vehicles than animals, which was not observed in the main effect. On the subordinate level, the effect was even reversed, showing higher accuracy (but not reaction times) for the animate category (which explains the missing main effect of animacy for accuracy). This shows that the animacy effect (better performance for vehicles) was mainly driven by basic level categorization. Previous studies investigating animacy effects in rapid categorization partly contradict our findings, as they found a small but consistent advantage for animal categories over vehicle categories [31], [32]. Another study [54], however, did not find a difference between animal and vehicle categories for superordinate rapid object detection. This agrees with our results, as performance was the same for both categories on the superordinate level. Rapid object categorization studies most often use the superordinate level of categorization and very heterogeneous stimulus sets, including many kinds of animals (mammals, fish, birds, and insects) and vehicles (cars, planes, bicycles, and ships). Although the stimulus set of the current study had less variable visual features across categories, the results were similar to previous studies.

These findings support the existence of different processing mechanisms for animate and inanimate categories. The question arises why the advantage of vehicles occurs only at the basic level. The effect might be a result of inappropriate selection of categories at the basic level. If this is the case, different processing mechanisms are engaged just because of a difference in level. However, categories were carefully chosen and perceptual similarity between object categories was ensured to make them as comparable as possible. Thus, all objects had roughly the same shape and retinal size. It was especially important for the basic level to provide similar difficulty levels between the two categories that were tested against each other (i.e. dog/cat and car/bus). Therefore, we can rule out that another category level than basic level was tested. One explanation for a vehicle advantage may be that animate objects engage different neural circuits for processing than inanimate objects [13], which might influence the categorization predominantly on the basic level. It has been previously shown that for superordinate categorization coarse information about an object is sufficient to differentiate between two categories [5]. For basic level categorization, more detailed visual information is necessary, which leads to a recruitment of further object-selective brain regions. Fine grained analysis takes longer and because animate and inanimate categories might be processed in distinct neural circuits, an additional time consuming processing step might be necessary for the animal category.

The *sensory/functional account* potentially gives an explanation of how the brain might solve the animal/non-animal distinction. This account is based on the assumption that objects are represented regarding their information content and functionality [35], [36]. It assumes that recognition performance for manipulable objects (e.g. tools) is enhanced due to the fast retrieval of action related circuits, which results in a “non-living identification advantage” [24]. Based on this account, *non-manipulable* artifacts (e.g. vehicles) depend more on their visual information than on their function, which might result in a loss of the non-living advantage. McMullen and colleagues [24] confirmed this assumption and found a disadvantage for non-manipulable artifacts compared to living things. Other studies challenged this idea and found neither an advantage nor a disadvantage for vehicles over animals at the superordinate level [54]. The results of the current experiment speak against the *sensory/functional account*. Reaction times to vehicles were faster than to animals, contra to our expectations. One possibility might be that we interact with vehicles more regularly in daily life and are therefore more trained to react to the category “vehicle” than to the category “animal”. Another reason might be based on common features of the object categories, corresponding to the *correlated feature theory* [55], [56]. This account addresses *correlated features* that often co-occur within an object category (e.g. has eyes, can see). According to this theory, living objects contain more similar features than non-living objects, and it should thus be more difficult to distinguish between them [57]. This would offer an explanation for the reduced processing speed for the animal category presented in this study. Features of objects that are necessary for categorization at a certain level, may engage different cognitive processing steps. If these features 1) differ between animate and inanimate categories and 2) differ between levels, a different behavioral outcome between animate and inanimate categories at the different levels of abstraction is expected. Features that need to be evaluated to differentiate a dog from a cat and a car from a bus may need longer processing time for animate categories due to higher similarity between dog/cat than car/bus [57]. Dogs and cats may share more common features (e.g. has eyes, can see; has legs, can walk) than cars and busses (e.g. has wheels, can drive – but car: drive myself; bus: has driver). Thus, cars and busses might be more easily discriminated due to fewer shared features than cats and dogs. From the current experiment it is not possible to draw further conclusions about the influence of shared visual features. An additional experiment that manipulates and controls for visual features would be necessary to prove this assumption. Another explanation may be a figure-ground segmentation advantage for vehicles. Vehicles are composed of geometric shapes with clear edges, which are easier segmented than animal objects with organic shapes with a flowing and curving contour. Therefore, perceptual processing of vehicles might be faster, leading to faster reaction times.

On the subordinate level, reaction times and error rates increase because more information about the single object is required and even finer grained information processing needs to be conducted. This may equalize the processing time of subordinate level animals (e.g. Siamese cat vs. Persian cat) and vehicles (e.g. Jeep vs. estate car) and make it even more difficult to categorize subordinate vehicles.

### Background of Objects

Contextual information is believed to facilitate object processing [58]. Any mismatch between object and background would, thus, lead to reduced facilitation effects. We hypothesized that an inverted background would inhibit rapid object categorization. The experiments showed that background inversion failed to affect performance. Subjects responded as accurately and as quickly to objects on an inverted background as to objects presented on an upright background. However, we also observed better performance for categorization of objects presented on a gray background than for objects presented on a complex background.

It is puzzling that we failed to show an effect of an inverted background on object categorization. Studies have shown that object/context incongruencies impact performance and produce higher error rates and slower reaction times in categorization tasks [41], [40]. There is evidence that semantic incongruence of object and context impair performance [38], [40]. This is true even for short exposure durations and speeded response times, indicating parallel processing of object and context information. Other experiments have demonstrated that manipulation of color [59], proportions [41] and orientation [43] in scenes hamper the recognition of a target object. We failed to find impaired object categorization with an inverted background. According to Rieger et al. [43], orientation effects are strongest if the context is rotated by 90°. They showed that rotation by 180° has a weaker effect in natural scene discrimination and suggest some “orientation compensation” mechanisms that allow faster processing of inverted scenes than for intermediate rotation angles. Only inclined orientation conflicts between object and background may affect behavior and inversion by 180° can be compensated for. Nevertheless, if the background information affects object processing, one would expect at least minor effects of inversion. One possibility is that in the current task the background information is redundant and not necessary to solve the task. Studies showing an influence of context on object processing found only small effects [40]. In these studies, objects were isolated and pasted on backgrounds, similar to our stimuli. However, the manipulation of background orientation is different from context manipulations used in other rapid categorization studies [32], [40]. In scene inversion, high-level visual information is modified, while low-level cues are kept constant [60]. This may lead to the conclusion that context effects derive from simple natural scene statistics, which were not changed in this study. In other words, if the nature of context effects depends on low- and mid-level information, then scene inversion should have no effect on categorization processes. On the other hand, local information cues may vary between objects on the same upright and inverted background. Thus, global low- and mid-level information is constant for upright and inverted images but local stimulus features may differ. This may affect object segmentation processes in both background conditions. As no effect between upright and inverted scenes was found, it is unlikely that these different local features have a strong impact on the categorization process. We cannot rule out that the missing context effect in our study results from inappropriate stimulus material. The objects were cropped out and pasted on an extra background. Although pasting effects between object and context were controlled for, the object was always in the foreground. It is possible that this “pop out” of the object minimized the effect of the scene inversion [61]. This may lead to the question of the strength of context effects.

We found an advantage for categorizing objects on a gray background relative to objects within scenes. Previous experiments which compared isolated objects to objects embedded in natural scenes also found that complex backgrounds affect categorization negatively [38] and challenge the idea of a general facilitation effect for congruent context [62]. One reason for better performance for isolated objects over objects with context might be that the figure-ground segmentation of the object is easier and faster. Due to less visual information and a lack of distractor elements in the background, the object categorization is improved. Thus, objects on a homogenous gray background are generally recognized more accurately and quickly than objects embedded in a congruent background [61].

The accuracy for categorizing objects with gray vs. complex background information was dependent on the level of categorization (in contradiction to reaction times, where facilitation for the gray background was observed at each level). On the subordinate level, subjects responded less accurately to objects with a complex background than to objects on gray background. Thus, the subordinate level was affected more strongly by additional background information than the other levels. This is probably caused by the higher processing demands required to categorize objects at the subordinate level and the resulting greater vulnerability to surrounding information. Most studies used the superordinate or basic level to investigate contextual effects [40], [62]. Joubert et al. [40], for example, found an advantage of objects on a gray background for reaction times but not for accuracy. As they tested the superordinate level only, their results are in accordance with the results presented here. As we also tested for background effects on the basic and subordinate levels, here we provide additional information about the strength of the influence of complex background information on object categorization. The subordinate level particularly is more affected by complex backgrounds, likely because higher processing demands are required to extract fine grained object information.

Animals were more influenced by additional background information than vehicles. Reaction times were significantly longer for the complex background condition than for the gray background condition for animals. As mentioned before, animal categories may share more common features than inanimate categories and, thus, require higher processing demands [57]. We consider this finding to be an explanation for the greater influence of background for animate categories if additional background information is presented. A greater effect of background for animal categories may arise from the scene information itself. Each object was pasted on a semantically congruent background, resulting in different scenes for each category (e.g. cars on streets, dogs in gardens). Thus, the animal related scene may require, in combination with animals, more time consuming processing. However, the level of context was not manipulated, which makes a systematic background-driven effect difficult to prove.

In summary, this study shows that the contextual information surrounding an object has an influence on categorization processes, which hamper object segmentation and, thus slow down object categorization. Effects of object-background incongruencies strongly depend on the factor that is manipulated (low- or high-level information) and likely also on the quality of the available information.

### Caveats

We termed the manipulations of this study “top-down” and each of them clearly has top-down processing components. However, we are aware that not only top-down mechanisms can account for the effects that were observed. Image statistics, such as spatial frequencies, influence categorization performance in a bottom-up fashion. Visual features and spatial frequencies differ between categories, which makes them inherently dissociable [33], [34], [60], [63], [64]. In the design of the current study, these confounds could not be completely controlled for, and such a limitation should be kept in mind when interpreting the results. Furthermore, physical dimensions and higher cognitive functions likely interact, such that low-level changes influence top-down processes [65].

Our interpretation of the results assumes that appropriate categories, levels of abstraction and context conditions were chosen. The concept of different category levels is not standardized and varies between studies. This hampers the interpretation of results and comparisons with other experiments. Studies investigating the influence of the cognitive components of object processing generally employ tasks that include imagination or naming of objects, using word or picture stimuli. Studies investigating the question of perceptual object processing rather use rapid categorization tasks (with limited object presentation time and button press responses). However, the overlap between studies investigating perceptual vs. cognitive object processing stages remains insufficient. Given that task requirements (go/no-go, match/mismatch or two-alternative forced choice task), stimuli (complex scenes, isolated objects, line drawings) and response criteria (button press or verbal response) tend to differ between studies, the different outcomes of these studies are not surprising. One interpretation of ambiguous findings regarding the animate/inanimate advantages might be the fact that inconsistent object categories at distinct levels of categorization were used. This could lead to different results. Nevertheless, further research needs to be conducted in order to amplify the understanding about the behavioral correlates of object categorization.

One further point to consider is the sometimes different outcome of accuracy and reaction time data. We interpreted accuracy as reflecting the difficulty of the task. This might arise from, for example situations where more information is needed to categorize an object on the subordinate than at the superordinate level. The additional amount of information needed increases the difficulty of categorization. Alternatively, due to higher similarity between categories at the subordinate level, misinterpretation of one category for the other can lead to higher error rates. Our interpretation of the reaction time data is different than our interpretation of the accuracy data. In our opinion, reaction times reflect the processing speed in the brain. If for two task conditions distinct networks with different processing time are engaged, different reaction times with similar accuracies may be observed. The dissociation between the response qualities may shed light on the underlying brain processes, but it has to be treated with caution. To our knowledge there is no proof for a neural correlate of accuracy and reaction times, respectively. Most often both processes work together and are difficult to disentangle.

### Conclusions

The current study investigated the influence of different top-down manipulations on rapid object categorization. We found that 1) objects are processed according to coarse-to-fine grained information (no basic level advantage present), 2) a benefit for vehicles at the basic level and for animals at the subordinate level, and 3) deteriorated categorization of objects presented on a complex background in comparison to a gray background, with the strongest effect at the subordinate level. We conclude that object categorization effects depend highly on the level and the type of category.
